# Computational Approaches to Facilitate Epitope-Based HLA Matching in Solid Organ Transplantation

**DOI:** 10.1155/2017/9130879

**Published:** 2017-02-12

**Authors:** Kirsten Geneugelijk, Jeroen Wissing, Dirk Koppenaal, Matthias Niemann, Eric Spierings

**Affiliations:** ^1^Laboratory of Translational Immunology, University Medical Center Utrecht, Utrecht, Netherlands; ^2^PIRCHE AG, Berlin, Germany

## Abstract

Epitope-based HLA matching has been emerged over the last few years as an improved method for HLA matching in solid organ transplantation. The epitope-based matching concept has been incorporated in both the PIRCHE-II and the HLAMatchmaker algorithm to find the most suitable donor for a recipient. For these algorithms, high-resolution HLA genotype data of both donor and recipient is required. Since high-resolution HLA genotype data is often not available, we developed a computational method which allows epitope-based HLA matching from serological split level HLA typing relying on HLA haplotype frequencies. To validate this method, we simulated a donor-recipient population for which PIRCHE-II and eplet values were calculated when using both high-resolution HLA genotype data and serological split level HLA typing. The majority of the serological split level HLA-determined* ln(PIRCHE-II)*/*ln(eplet)* values did not or only slightly deviate from the reference group of high-resolution HLA-determined* ln(PIRCHE-II)*/*ln(eplet)* values. This deviation was slightly increased when HLA-C or HLA-DQ was omitted from the input and was substantially decreased when using two-field resolution HLA genotype data of the recipient and serological split level HLA typing of the donor. Thus, our data suggest that our computational approach is a powerful tool to estimate PIRCHE-II/eplet values when high-resolution HLA genotype data is not available.

## 1. Introduction

Alloimmunity due to Human Leukocyte Antigens (HLA) mismatches between donor and recipient significantly impairs graft survival after solid organ transplantation [1–3]. The risk on graft failure is significantly associated with the number of HLA mismatches [1, 4]. Therefore, some allocation policies prefer deceased donors with zero mismatches at HLA-A, HLA-B, and HLA-DR, whereas others select deceased donors based on the number mismatches at these loci [5].

Although the number of HLA mismatches is a potent predictor of transplant outcome, not every HLA mismatch will have an equal effect on graft failure [6, 7]. Cumulating evidence suggest that some HLA mismatches may induce alloimmunity, whereas others are well-tolerated [6, 7]. This high variability in permissibility might be due to differences in the antigenic load between different donor-recipient couples [8, 9]. Each HLA antigen expresses a unique combination of epitopes, but some of these individual epitopes may be shared between different HLA antigens [8]. These shared epitopes will not induce alloimmunity, whereas those epitopes that are mismatched between donor and recipient may induce alloimmunity. Thus, quantifying the antigenic load (i.e., the number of epitope mismatches) between donor and recipient instead of counting the number of HLA mismatches may be a better approach to predicting transplant outcome [9–12]. This concept of epitope-based HLA matching is an alternative method to define the most suitable HLA mismatch for each patient, thereby reducing the risk on donor-specific HLA antibody formation after transplantation and graft failure.

Two in silico methods, HLAMatchmaker and PIRCHE-II, have incorporated the epitope-based HLA matching concept in their algorithm to find the most suitable donor for a recipient. HLAMatchmaker determines differences in B-cell epitopes between donor and recipient to estimate the risk of graft failure [13–15]. These B-cell epitopes, designated as eplets, are groups of polymorphic amino acid positions on the three-dimensional molecular surface of HLA to which HLA antibodies can be formed [13–15]. The PIRCHE-II algorithm determines differences between donor and recipient in their HLA-derived T-helper epitopes to estimate the risk of transplant outcome [16]. These T-helper epitopes, designated as PIRCHE-II (Predicted Indirectly ReCognizable HLA Epitopes presented by HLA-DRB1), are involved in the production of HLA-specific IgG antibodies [17–19], as T-helper epitopes are required for B-cell activation and IgM-to-IgG isotype switching [20, 21].

To be able to identify the unique set of donor and recipient HLA epitopes, identification of the exact polymorphisms in donor and recipient HLA is required. Low-resolution HLA typing is, however, not sufficient to identify these polymorphisms, as low-resolution HLA typing can cover numerous HLA alleles at high-resolution HLA level. Thus, low-resolution HLA typing will lead to an ambiguous epitope definition. Therefore, two-field resolution HLA genotype data of both donor and recipient is preferably required to unambiguously determine the HLA compatibility between donor and recipient at epitope level. Indeed, both HLAMatchmaker and the PIRCHE-II algorithm require high-resolution HLA genotypes of both donor and recipient as input for their algorithm. Although HLA genotyping methodologies have improved over the last few years and high throughput NGS technology became available, the quick availability of reliable high-resolution HLA genotype data remains challenging. High-resolution HLA genotyping is especially for deceased donors hardly feasible, as time is a major limiting factor for deceased organ transplantation.

Instead of high-resolution HLA genotyping of donor and recipients, also alternative approaches can be used to facilitate epitope-based HLA matching. In the present study we describe a computational method to perform epitope-based HLA matching using serological split level HLA typing as input. In this computational method, the most likely high-resolution HLA genotypes that correspond to a serological split level HLA typing are identified using HLA haplotype frequency tables. For all of these high-resolution HLA genotypes, a PIRCHE-II and eplet value can be calculated, which can subsequently be weighed against the normalized frequency of the pair of HLA haplotypes in the general population. To test whether the risk estimation alters when using our computational approach, we calculated the PIRCHE-II/eplet values when using serological split level HLA typing (designated as observed PIRCHE-II/eplet values) and compared these values with the PIRCHE-II and eplet values when using high-resolution HLA genotype data (designated as reference PIRCHE-II/eplet values).

## 2. Materials and Methods

### 2.1. Generation of the Representative Recipient Population

To model a representative recipient population, all HLA genotypings (*n* = 4,579) that were performed at the University Medical Center Utrecht between January 2009 until July 2016 were extracted from the lab system. HLA genotypings were performed at different resolution levels and for different loci. High-resolution HLA genotypings were performed by SBT (before 2014; SBT kit, genDX, Utrecht, The Netherlands) or NGS (2014 and later; NGSGo, genDX, Utrecht, The Netherlands) whereas PCR-SSO (One Lambda) was used for lower-resolution HLA genotyping. HLA genotypes that did not have a fully unambiguous high-resolution HLA typing for HLA-A, HLA-B, HLA-C, HLA-DRB1, and HLA-DQB1 were excluded from the population. A total of 2,373 typings had an unambiguous high-resolution HLA genotype data for HLA-A, HLA-B, HLA-C, HLA-DRB1, and HLA-DQB1. These typings were used to simulate a representative recipient population.

### 2.2. Generation of the Virtual Donor Population

A virtual Caucasian donor population consisting of 10 million individuals was modeled using HLA haplotype frequency tables from the National Marrow Donor Program of 2007 [22]. The HLA haplotype frequency tables are available via https://bioinformatics.bethematchclinical.org/hla-resources/haplotype-frequencies/. To generate each individual of the 10 million individuals, two HLA haplotypes were randomly assigned to an individual. This assignment was based on the frequency of these HLA haplotypes within the HLA haplotype frequency tables. In this procedure sampling of HLA haplotypes was performed without replacement. Combined with the representative recipient population, this Caucasian population was subsequently used as a virtual donor population to form donor-recipient couples.

### 2.3. Generation of Donor-Recipient Couples

The virtual donor population and the representative recipient population were combined to form potential donor-recipient couples. Since random allocation will lead to a nonrepresentative distribution of HLA mismatches, donor-recipient couples were formed using the basic guidelines that are currently used for deceased kidney allocation at our local center. For each donor, a recipient was selected that had maximal 3 mismatches at HLA-A and HLA-B and maximal a single mismatch at HLA-DR. To this end, both the HLA genotypes of the representative patient population and the virtual donor population were converted into serological broad level HLA typing, which was used for matching. A donor was randomly selected from the virtual donor population and this donor was subsequently matched to a recipient. When for a certain donor-recipient combination four mismatches at HLA-A and HLA-B and/or two mismatches at HLA-DR were found, another recipient was selected for that donor until the matching criteria were met for a given donor-recipient couple. This procedure was followed until a virtual donor was found for each recipient of the representative recipient population.

This method has resulted in 2,373 donor-recipient couples. A total of 11 donor-recipient couples had zero mismatches, 165 had a single mismatch, 571 had two mismatches, 890 had three mismatches, and 736 had four mismatches at these loci. A total of 2,195 mismatches were at HLA-A, 2,823 at HLA-B, and 1,903 at HLA-DR.

Serological split level HLA typing (observation group) and two-field resolution HLA genotypes (reference group) of these donor-recipient couples were used to calculate the number of PIRCHE-II and eplets, as described below. Although donor-recipient couples were formed based on HLA-A, HLA-B, and HLA-DR only, the complete five loci-haplotypes (HLA-A, HLA-B, HLA-C, HLA-DR, and HLA-DQ) were used to calculate the PIRCHE-II and eplet values. PIRCHE-II and eplet values were also calculated when HLA-C or HLA-DQ was removed from the serological split level HLA-A, HLA-B, HLA-C, HLA-DR, and HLA-DQ typing or when using two-field resolution HLA genotype data of the recipient and serological split level HLA typing of the donor as input for the algorithms.

### 2.4. The Use of Serological Split Level HLA Typing to Identify Potential High-Resolution HLA Genotypes

For the serological split level HLA typings of the 2,373 donor-recipient couples, a high-resolution extrapolation method was used to identify all possible high-resolution HLA genotypes that correspond to each serological split level HLA typing. To this end, HLA haplotype frequency tables from the National Marrow Donor Program from 2007 and 2011 were both used separately to identify all potential high-resolution HLA genotypes from a serological split level HLA typing [22, 23].

For every given serologic split level HLA typing, the extrapolation algorithm started with setting up all potential two-field resolution HLA haplotype pairs (HLA-A, HLA-B, HLA-C, HLA-DRB1, and HLA-DQB1) that yield the given input serological split level HLA typing. After mapping antigen/allele names for each serologic value, the haplotype frequency table was filtered for matching haplotypes. When no matching high-resolution HLA haplotype was found for a given serological split level HLA typing or subsets thereof, the selection criteria were broadened by removing the link between loci in a step-wise manner. The following order of HLA loci linkage removal was used: [i] A-B-C-DRB1-DQB1, [ii] A-B-C | DRB1-DQB1, [iii] A | B-C | DRB1-DQB1, [iv] A | B-C | DRB1 | DQB1, and [v] A | B | C | DRB1 | DQB1. It has to be noted that mode [v] is equivalent to using allele frequencies for the prediction of each individual locus. Supplementary material 1 in Supplementary Material available online at https://doi.org/10.1155/2017/9130879 shows an example of the linkage breakdown between HLA loci.

Since multiple high-resolution HLA genotypes may correspond to a single serological split level HLA typing, a list of potential high-resolution HLA genotypes was generated for both donor and recipient. For each typing, a frequency was calculated by multiplying both haplotypes' frequencies. The resulting absolute frequencies were normalized within the set of likely high-resolution HLA genotypes for the given input.

This method was applied to both donors and recipients. For all these potential high-resolution HLA genotypes of donor and recipient, the PIRCHE-II and eplets values were calculated as described below. The obtained PIRCHE-II values were subsequently weighted by multiplying the normalized frequency of a certain high-resolution HLA genotype of the recipient with the normalized frequency of a certain high-resolution HLA genotype of the donor. Finally, all weighted PIRCHE-II values were summed up. The same was applied to the eplet values. Thus, the used method takes all potential high-resolution HLA genotypes that are present in the HLA haplotype frequency table into account which corresponds to a certain serological split level HLA typing. By using all these genotypes and by weighing the epitope values, multiple imputation is used to minimize bias towards common HLA genotypes.

In this study, the HLA haplotype frequency tables of the Caucasian population (2007) and the European-Caucasian population (2011) were used in this study to determine all potential high-resolution HLA genotypes.

### 2.5. Identification of PIRCHE-II

For both the two-field resolution HLA genotypes (designated as reference group) and the serological split level HLA typings (designated as observation group), the number of PIRCHE-II was determined for each donor-recipient couple as described previously [17]. Briefly, the nonameric binding cores of mismatched-HLA derived peptides to recipient HLA-DRB1 (PIRCHE-II) were predicted using the NetMHCIIpan 3.0 algorithm. Peptides that had an IC_50_ < 1000 nM were considered as relevant HLA-DRB1 binders. These relevant HLA-DRB1 binders were only classified as a PIRCHE-II when the amino acid residues of the nonameric binding cores were not present in the amino acid sequence of recipient HLA. The PIRCHE algorithm is available via https://www.pirche.org/.

### 2.6. Identification of HLAMatchmaker Eplets

For both the two-field resolution HLA genotypes and the serological split level HLA typing, the number of mismatched eplets was determined for each donor-recipient couple using eplet definitions as provided in HLAMatchmaker algorithm version 2.1 (available via http://www.epitopes.net/). Mismatched eplets were defined as eplets that were present in donor HLA but absent in recipient HLA. Via interlocus HLA comparisons the number of mismatched eplets was determined.

### 2.7. Comparison between Serological Split Level HLA Typing and Two-Field Resolution HLA Genotype Data

When using serological split level HLA typing as input for the algorithm, PIRCHE-II and eplet values were obtained for 2,319 donor-recipient couples (97.7%) when using the HLA haplotype frequency tables of 2007 or for 2,369 donor-recipient couples (99.8%) when using the HLA haplotype frequency tables of 2011.

The number of epitopes and alloreactivity are unlikely correlated in a linear fashion; increases from 1 epitope to 10 epitopes are likely to have a higher impact than increases from 200 epitopes to 210 epitopes and the effects are likely to plateau at some point. As such we assume an inverse logarithmic effect of epitope numbers on alloreactivity and we converted the PIRCHE-II and eplet numbers into the natural logarithm thereof. These transformed PIRCHE-II and eplet values were subsequently used to identify differences between the two-field HLA genotype-determined PIRCHE-II/eplet values, designated as “reference PIRCHE-II/eplet values,” and the serological split level HLA typing-determined PIRCHE-II/eplet values, designated as “observed PIRCHE-II/eplet values.” The delta between the observed and reference PIRCHE-II/eplet values was calculated by subtracting the log-transformed reference PIRCHE-II/eplet values from the log-transformed observed PIRCHE-II/eplet values. A delta of >+1 or <−1 was considered a high deviation, as it represents a single unit of* ln(PIRCHE-II)* or* ln(eplet)* difference and thus a significant over- or underestimation of the hazard in graft failure. For all tested settings, we calculated the delta that corresponded to the 50% percentiles, 75% percentiles, 95% percentiles, 99% percentiles, and 99.9% percentiles to test the reliability of our estimations. For example, for the 75% percentile we calculated to which delta 25% of the values above the median stretched and to which delta 25% of the values below the median stretched. A similar approach was used for the other percentiles.

## 3. Results

### 3.1. Reliability of Epitope Matching When Using Serological Split Level HLA Typing

For all donor-recipient couples we calculated the PIRCHE-II and eplet values when using two-field HLA genotype data and serological split HLA typing as input for the module. The PIRCHE-II/eplet values determined based on two-field resolution HLA genotypes were designated as “reference PIRCHE-II/eplet values,” whereas the PIRCHE-II/eplet values determined based on serological split HLA typing were designated as “observed PIRCHE-II/eplet values.”

**Figure 1:**
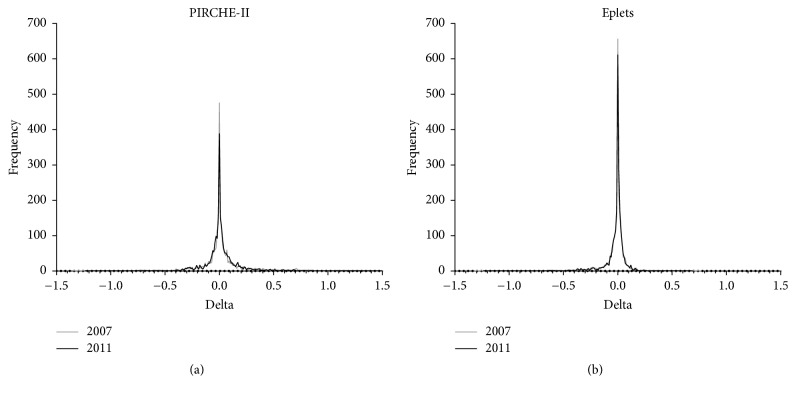
Deviation of the observed* ln(PIRCHE-II)/ln(eplet)* values from reference* ln(PIRCHE-II)/ln(eplet)* values when using HLA haplotype frequency tables from 2007 or from 2011. For both* ln(PIRCHE-II)* (a) and* ln(eplet)* (b), the observed values do not deviate or only slightly deviate from the reference values. The use of haplotype HLA frequency tables from 2007 (gray line) or from 2011 (black line) resulted in similar deviations.

First we determined whether the use of serological split level HLA typing as input led to different PIRCHE-II and eplet values compared to the use of two-field resolution HLA genotype data as input. To this end, the delta between the observed* ln(PIRCHE-II)* and* ln(eplet)* values and the reference* ln(PIRCHE-II)* and* ln(eplet)* values was calculated (Figure 1). When using the frequency tables of 2007, 20.5% of observed* ln(PIRCHE-II)* values did not show deviation from the reference* ln(PIRCHE-II)* values (Figure 1(a)). Furthermore, 72.8% of the observed* ln(PIRCHE-II)* values deviated maximal 0.1 from the reference* ln(PIRCHE-II)* values. When using the frequency tables of 2007, 28.3% of observed* ln(eplet)* values did not deviate from the reference* ln(eplet)* values and 87.7% deviated maximal 0.1 (Figure 1(b)). These data indicate that majority of the observed* ln(PIRCHE-II)* and* ln(eplet)* values do not deviate or only slightly deviate from reference* ln(PIRCHE-II)* and* ln(eplet)* values.

For both PIRCHE-II and the eplets, similar results were obtained when using the frequency tables of 2011 instead of using the frequency tables of 2007. When using the frequency tables of 2011, 16.4% of observed* ln(PIRCHE-II)* values did not show deviation from the reference* ln(PIRCHE-II)* values and 73.2% deviated maximal 0.1 (Figure 1(a)). When using the frequency tables of 2011, 25.8% of observed* ln(eplet)* values did not deviate from the reference* ln(eplet)* values and 88.4% deviated maximal 0.1 (Figure 1(b)). This observation suggests that the use of these more recent frequency tables in our computational method does not improve or worsen the observed PIRCHE-II/eplet values.

**Figure 2:**
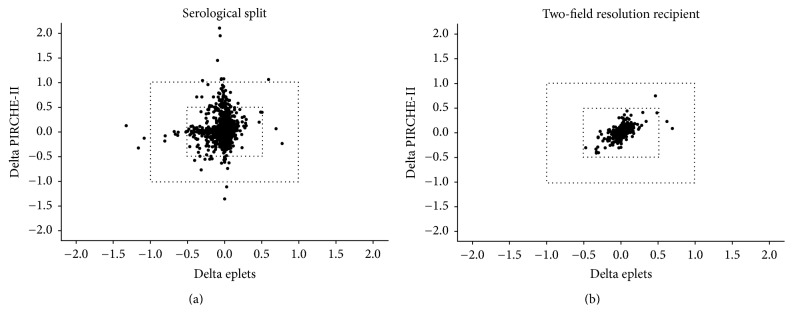
Comparison of the delta in* ln(PIRCHE-II)* and the delta in* ln(eplet)*. (a) When using serological split level HLA typing of both donor and recipient, the majority of the donor-recipient couples had a comparable* ln(PIRCHE-II)* and* ln(eplet)* delta. For 13 donor-recipient couples, the observed* ln(PIRCHE-II)* or* ln(eplet)* values deviated substantially (>+1 or <−1) from the reference* ln(PIRCHE-II)* or* ln(eplet)* values. (b) When using two-field resolution HLA genotype data of the recipient and serological split level HLA typing of donor the deviation between the observed* ln(PIRCHE-II)/ln(eplet)* values and the reference* ln(PIRCHE-II)/ln(eplet)* values substantially diminished. The dashed squares indicate the delta<0.5 and delta<1 borders.

Next, we investigated whether the delta in* ln(PIRCHE-II)* values differed from the delta in* ln(eplet)* values. To this end, we plotted the delta for* ln(PIRCHE-II)* against the delta for* ln(eplet)*. When using serological split level HLA typing of both donor and recipient, the majority of the donor-recipient couples had a comparable* ln(PIRCHE-II)* and* ln(eplet)* delta (Figure 2(a)). However, two donor-recipient couples (0.08% of total) had a* ln(PIRCHE-II)* delta below −1, eight donor-recipient couples (0.34% of total) had a* ln(PIRCHE-II)* delta above +1, and three donor-recipient couples (0.13% of total) had a* ln(eplet)* delta below −1. These data suggests that the PIRCHE-II value is more often overestimated using the extrapolation approach, whereas the eplet value is more often under-estimated using the extrapolation approach. For all the donor-recipient couples who had a high deviation in the* ln(PIRCHE-II)* or* ln(eplet)* values, the corresponding* ln(eplet)* or* ln(PIRCHE-II)* values respectively were within the −1 and +1 range. This observation indicates that an increased delta in* ln(PIRCHE-II)* is not associated with an increased delta in* ln(eplet)* and vice versa.

### 3.2. Removal of HLA-C or HLA-DQ and Reliability of Estimation

**Figure 3:**
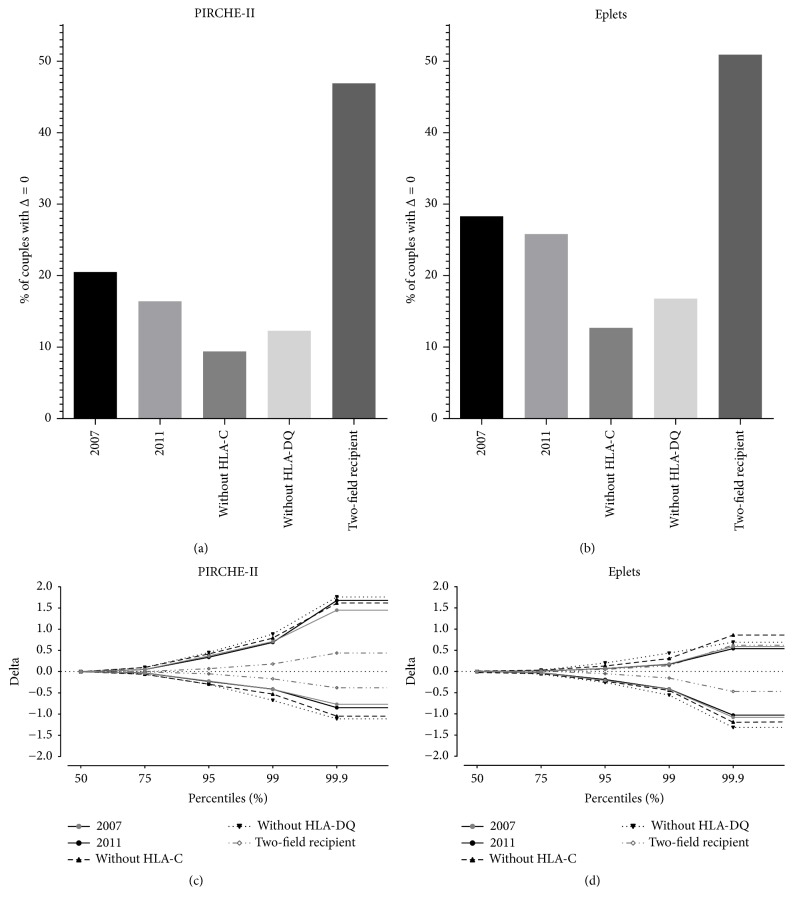
The reliability of the PIRCHE-II and eplet estimations in different settings. The percentage of typing with a delta of zero between the observed and the reference values was plotted for* ln(PIRCHE-II)* (a) and* ln(eplet)* (b). For both* ln(PIRCHE-II)* and* ln(eplet)*, the percentage of typing with a delta of zero was diminished when HLA-C or HLA-DQ was omitted from the typing. The highest percentage was observed when using two-field HLA genotype data of the recipient and serological split level typing of the donor. The different percentiles observed in the different settings were plotted for* ln(PIRCHE-II)* (c) and for* ln(eplet)* (d). The dashed horizontal lines in (c) and (d) indicate a delta of zero.

HLA-B is in strong linkage disequilibrium with HLA-C and HLA-DRB1 is in strong linkage disequilibrium with HLA-DQB1. This strong linkage disequilibrium suggests that removal of HLA-C or HLA-DQ from the serological split level HLA typing might only limitedly affect the observed PIRCHE-II/eplet values and thus the delta between the observed and the reference* ln(PIRCHE-II)*/*ln(eplet)* values. To investigate the effect of HLA-C or HLA-DQ on the PIRCHE-II and eplet estimations, we removed HLA-C or HLA-DQ from the serological split level HLA typing that was used as input for both PIRCHE-II and HLAMatchmaker.  Figure 3 shows the percentage of donor-recipient couples with a delta of zero between the observed and the reference* ln(PIRCHE-II)* and* ln(eplet)* values. When omitting HLA-C or HLA-DQ from the input, the percentage of donor-recipient couples who had a* ln(PIRCHE-II)* delta of zero dropped from 20.5% to 9.4% or 12.3% for omitting HLA-C or HLA-DQ, respectively (Figure 3(a)). For the eplets, similar results were obtained. The percentage of donor-recipient couples who had an* ln(eplet)* delta of zero dropped from 28.3% to 12.7% or 16.8% for omitting HLA-C or HLA-DQ, respectively (Figure 3(b)).

To investigate the reliability of the epitope estimation when omitting HLA-C or HLA-DQ, we plotted the* ln(PIRCHE-II)* and* ln(eplet)* delta values for different percentiles (50%, 75%, 95%, and 99.9%). When omitting HLA-C or HLA-DQ from the serological split level HLA typing, the delta values for the higher percentiles slightly increased compared to delta values obtained from serological split level typing with HLA-C and HLA-DQ (Figures 3(c) and 3(d)). These observations indicate that removal of HLA-C or HLA-DQ from the serological split level HLA typing diminishes the reliability of the observed PIRCHE-II/eplet values.

### 3.3. Effect of Higher Resolution Typing of Recipient

Since high-resolution HLA genotyping is a time-consuming method, high-resolution HLA genotyping of deceased donors is hardly feasible using the currently available typing methodologies. However, in most cases, high-resolution HLA genotyping of the recipient is possible. Therefore, we investigated whether a higher resolution HLA genotyping of the recipient may improve the reliability of the PIRCHE-II and eplet estimations. To this end, we used two-field resolution HLA genotype data of the recipient and serological split level HLA typing of the donor as input for the PIRCHE-II and the HLAMatchmaker algorithm. The percentage of donor-recipient couples who had a* ln(PIRCHE-II)* delta of zero increased from 20.5% to 46.9% when using two-field resolution genotype data of the recipient (Figure 3(a)). For the* ln(eplet)*, this percentage of donor-recipient couples who had a delta of zero increased from 28.3% to 50.9% (Figure 3(b)). The* ln(PIRCHE-II)* delta and the* ln(eplet)* delta diminished at all percentile values when using two-field resolution genotype data of the recipient instead of serological split HLA typing (Figures 3(c) and 3(d)). These data indicate that higher resolution genotyping of the recipient decreases the delta between the observed and the reference* ln(PIRCHE-II)* and* ln(eplet)* values and thus increases the reliability of the observed PIRCHE-II and eplet values. The reliability of the estimation was especially improved for PIRCHE-II and to a substantial but lesser extend for eplets. For all different settings tested, the most reliable PIRCHE-II/eplet estimation was achieved when using two-field resolution genotype data of the recipient and serological split level HLA typing of the donor.

When comparing the delta in* ln(PIRCHE-II)* values with the delta in* ln(eplet)* values for the two-field resolution recipient setting, all donor-recipient couples had comparable* ln(PIRCHE-II)* and* ln(eplet)* delta values (Figure 2(b)). For all couples, no outliers for* ln(PIRCHE-II)* and* ln(eplet)* were observed. Thus, our data suggest that two-field resolution HLA genotyping of the recipient further improve the reliability of the PIRCHE-II and eplet estimation.

## 4. Discussion

Several studies have shown that the number of PIRCHE-II and eplets are related to the clinical outcome after solid organ transplantation [9–12]. To select donors in an epitope-based manner, high-resolution genotyping of both donors and recipients is currently required. However, high-resolution HLA genotyping is often not feasible, particularly for deceased-donor organs. In this study we describe and validate a computational method to facilitate epitope-based HLA matching using low-resolution HLA typing.

In the present study we provide a methodology for using low-resolution serological split level HLA data to reliably estimate the PIRCHE-II and eplet values for the majority of the tested donor-recipient couples (Figure 1). Most of the observed* ln(PIRCHE-II)* and* ln(eplet)* values do not deviate or only slightly deviate from the reference* ln(PIRCHE-II)* and* ln(eplet)* values (Figure 2(a)). These data suggest that additional high-resolution HLA genotyping may not be necessary for the majority of the donor-recipient couples when using our described computational approach. However, furthers validations in other datasets consisting of different ethnicities are required to identify for which donor-recipient couples additional high-resolution HLA genotyping is required and for which donor-recipient couples low-resolution HLA typing is sufficient.

We also showed that the PIRCHE-II/eplet estimation deteriorated or improved in different settings. The removal of HLA-C and HLA-DQ increased both the* ln(PIRCHE-II)* delta and the* ln(eplet)* delta (Figure 3), indicating that the PIRCHE-II/eplet estimation is deteriorated when HLA-C and HLA-DQ are omitted from the input. Based on these results, we suggest that HLA-C and HLA-DQ typing are a valuable addition when using epitope-based HLA matching algorithms. Two-field resolution HLA genotyping of the recipient in combination with serological split level HLA typing of the donor substantially improved the PIRCHE-II/eplet estimation, as reflected by the decreased delta between the observed and the reference* ln(PIRCHE-II)*/*ln(eplet)* values (Figures 2(b) and 3). Since time allows typing of the recipient at high- or even allelic resolution, the situation of a two-field resolution genotyped recipient and a serological split level HLA typed donor may be a feasible option in many cases and this will substantially improve the reliability of the PIRCHE-II/eplet estimation. This approach is now current practice in our center.

When comparing the PIRCHE-II results with the eplet results for the serological split level data, the delta for* ln(PIRCHE-II)* was more often positive than negative, whereas for* ln(eplet)* the delta was more often negative than positive (Figures 2(a), 3(b), and 3(c)). We also showed that a high deviation in* ln(PIRCHE-II)* values is not related to a high deviation in* ln(eplet)* values; some donor-recipient couples had a high* ln(PIRCHE-II)* delta and a small* ln(eplet)* delta and other donor-recipient couples had a high* ln(eplet)* delta and a small* ln(PIRCHE-II)* delta (Figure 2(a)). These observations suggest that serological epitopes (eplet) can more easily be determined with a serological split level HLA typing than T-helper epitopes (PIRCHE-II). In addition, these observations might also suggest that PIRCHE-II and eplets are indeed two separate entities, as already suggested in previous studies [17, 19].

Our study has several limitations. First, the virtual Caucasian donor population used in our study was constructed upon the HLA haplotype frequency tables from 2007. However, these HLA haplotype frequency tables from 2007 also formed the basis for our PIRCHE-II/eplet estimation approach and therefore may cause bias in our observations or overfitting of the results. When using the updated HLA haplotype frequency tables from 2011, similar observed PIRCHE-II/eplet values were obtained (Figure 1), indicating that the bias is limited. However, usage of the 2007 HLA haplotype frequency tables showed a slightly improved PIRCHE-II/eplet value estimation compared to the 2011 HLA haplotype frequency tables, which might be due to overfitting of the results. Second, by calculating a delta between natural logarithmic transformed data, the difference in the actual PIRCHE-II/eplet count may be masked for several donor-recipient couples. However, since the effect of the PIRCHE-II/eplet count on alloreactivity is likely natural logarithmic, calculating the delta using log-transformed data gives more insight into the alterations of the hazard on alloreactivity rather than differences in actual epitope count. Third, our validation is limited to the Caucasian population and can only limitedly be extrapolated for other ethnicities. Since the Caucasian HLA haplotype frequency tables were based on a large-scale dataset [22, 23], we used these frequency tables to estimate the PIRCHE-II/eplet values. The estimation of the PIRCHE-II/eplet values might less or more deviate when investigating different ethnicities. Considering all these study limitations, further studies, especially with different data sets and with different ethnicities, are required to validate our observations. Ultimately, fine-details on the donor ethnicity and the related frequencies, for instance, as documented by http://allelefrequencies.net [24], may further enhance the reliability of the extrapolations.

Our study showed that the observed PIRCHE-II and eplet values only limitedly deviate from the reference PIRCHE-II and eplet values in a quantitative manner; only differences in PIRCHE-II and eplet numbers were investigated in this study. However, although PIRCHE-II and eplet values do not differ between the observation group and the reference group, both calculations may correspond to different epitopes. Further analyses are required to identify whether the observed and reference calculations will have qualitative differences in epitopes.

To our knowledge this is the first study that uses this computational approach for epitope-based HLA matching algorithms. Other studies have been using the HLA haplotype frequency tables in a similar way but in different settings. For example, a similar approach as our approach has been used by Madbouly et al. for the imputation of high-resolution HLA genotypes from multilocus unphased genotypes with ambiguous or missing typing data [25]. In addition, the HLA Haplotype Validator described by Osoegawa et al. uses HLA haplotype frequency tables to extract all potential HLA haplotype constellations to identify potential errors in HLA genotyping [26]. Furthermore, HaploStats from the National Marrow Donor Program (available via http://www.haplostats.org/) also estimates the most likely high-resolution HLA genotypes, but without HLA loci linkage removal when no matching high-resolution HLA haplotype is found. Thus, imputation of data can only take place when the HLA haplotypes are present in the HLA haplotype frequency tables. For the HLAMatchmaker algorithm, HLA haplotype frequency tables have been used to identify the most frequently present HLA haplotype in the population that corresponds to a given low-resolution HLA typing. This most frequently HLA haplotype is subsequently used as input for the algorithm. By using this approach, other potential less-frequent HLA haplotypes that also fit with the given low-resolution HLA typing are excluded. In our multiple imputation approach, high-resolution HLA genotypes with reduced likelihood were taken along in the PIRCHE-II and eplet calculations. Since our approach does not exclude less likely HLA haplotypes, we believe that our approach is more reliable than selecting the highest frequent high-resolution HLA haplotypes that is present in the general population.

Our algorithm currently only handles serological split level HLA typing, whereas serological broad level HLA typing cannot be used as input for the algorithm. Likely, serological broad level HLA typing will further deteriorate our estimations and, consequently, is not preferred for an epitope-based HLA matching setting. Moreover, since serological split level HLA typing of both donors and recipients is currently mandatory according to the Eurotransplant guidelines [27], serological split level HLA typing is available for all donor-recipient couples and thus can be used in our approach. Extension of the computational method, for example, by providing genotype list string (GL string) [28] or adding NMDP allele codes [29] to the HLA typing input, might eventually further enhance the estimations of the PIRCHE-II/eplet values. Further studies are required to investigate whether addition of these functions will enhance the reliability of the estimations.

Our data show that although the PIRCHE-II/eplet value estimations are quite reliable, the estimations could be further improved. First, for a few donor-recipient couples, the PIRCHE-II/eplet values could not be calculated when using serological split level HLA typing, indicating that our approach cannot be used for a limited number of donor-recipient couples. One of the major improvements will be extension of the Next-Generation Sequencing-based genotype datasets that are used for establishing HLA haplotype frequency tables. As of November 2016, 11,553 HLA class-I alleles and 4,084 HLA class-II alleles are registered in the IMGT/HLA database 3.26 [30]. These high numbers of identified HLA alleles indicate that huge population HLA genotype datasets are required to reliably estimate the HLA haplotype frequencies. Indeed, a previous study has shown that the HLA haplotype frequencies are overestimated when small sample sizes are used [31]. This aspect stretches that more detailed information and reliable typing of different HLA haplotypes is required. Although the Caucasian HLA haplotype frequency table of the NMDP is based on the largest dataset, the sample size is still limited considering all the identified HLA alleles. This limited sample size might bias our results. Indeed, rare alleles are hardly present in the HLA haplotype frequency tables and, consequently, a rare allele among donor and recipients at high-resolution level can often not be identified using the current HLA haplotype frequency tables. Moreover, the NMDP HLA haplotype frequency tables are generally based on exon 2-3 for HLA class-I alleles and on exon 2 for HLA class-II alleles. Therefore, sharing of whole gene NGS sequencing-based high-resolution HLA genotyping may significantly improve the reliability of the HLA haplotype frequency tables and, thus, the reliability of our epitope-based matching estimations.

In conclusion, we have shown that the currently used extrapolation method is a powerful and reliable tool to estimate PIRCHE-II and eplet values. This method provides the opportunity to calculate PIRCHE-II and eplet values when using serological split level HLA typing and, thus, makes high-resolution HLA genotyping presumably redundant for the majority of the donor-recipient couples. When more next-generation sequencing-determined HLA genotype data will become available, HLA haplotype frequency tables will become more reliable and, consequently, the reliability of our epitope-based HLA matching estimations will be further improved.

## Supplementary Material

When no matching high-resolution HLA haplotype was found for a given serological split level HLA typing, the search criteria were broadened by removing an additional link between HLA loci. By removing an addition link, frequency calculations were adapted. A simplified example of the calculations when an additional link between HLA loci is removed is shown in Supplementary material 1.

## Abbreviations

HLA: Human Leukocyte Antigens
PIRCHE-II: Predicted Indirectly ReCognizable HLA Epitopes presented by HLA-DRB1.
