# Supplementary material for: Computational Approaches to Facilitate Epitope-Based HLA Matching in Solid Organ Transplantation
Source: J Immunol Res. 2017 Feb 12;2017:9130879. doi: 10.1155/2017/9130879 (PMC5329668; doi:10.1155/2017/9130879)
Supplement: Supplementary file 1 — When no matching high-resolution HLA haplotype was found for a given serological split level HLA typing, the search criteria were broadened by removing an additional link between HLA loci. By removing an addition link, frequency calculations were adapted. A simplified example of the calculations when an additional link between HLA loci is removed is shown in Supplementary material 1. [file 9130879.f1.pdf]

SUPPLEMENTARY MATERIAL

A. Serological split level input typing: **A1, A2, B8, B60, Cw7, Cw10, DR17, DR1, DQ2, DQ5**

B. HLA haplotype table including the haplotype frequencies

A*01:01	B*08:01	C*07:01	DRB1*03:01	DQB1*02:01	0.074
A*02:01	B*40:01	C*03:04	DRB1*13:02	DQB1*06:04	0.010
A*03:01	B*35:01	C*04:01	DRB1*01:01	DQB1*05:01	0.013
A*68:02	B*14:02	C*08:02	DRB1*13:03	DQB1*03:01	0.003

C. Potential well-defined haplotype pairs and their frequency in the HLA haplotype frequency tables:

**Haplotype 1**	**A*01:01**	**B*08:01**	**C*07:01**	**DRB1*03:01**	**DQB1*02:01**	**0.074**
Haplotype 2	A*02	B*40	C*03	DRB1*01	DQB1*05	not found
Haplotype 1	A*01	B*08	C*07	DRB1*03	DQB1*05	not found
Haplotype 2	A*02	B*40	C*03	DRB1*01	DQB1*02	not found
Haplotype 1	A*01	B*08	C*07	DRB1*01	DQB1*02	not found
Haplotype 2	A*02	B*40	C*03	DRB1*03	DQB1*05	not found
Haplotype 1	A*01	B*08	C*07	DRB1*01	DQB1*05	not found
Haplotype 2	A*02	B*40	C*03	DRB1*03	DQB1*02	not found
Haplotype 1	A*01	B*08	C*03	DRB1*03	DQB1*02	not found
Haplotype 2	A*02	B*40	C*07	DRB1*01	DQB1*05	not found
Haplotype 1	A*01	B*08	C*03	DRB1*03	DQB1*05	not found
Haplotype 2	A*02	B*40	C*07	DRB1*01	DQB1*02	not found
Haplotype 1	A*01	B*08	C*03	DRB1*01	DQB1*02	not found
Haplotype 2	A*02	B*40	C*07	DRB1*03	DQB1*05	not found
Haplotype 1	A*01	B*08	C*03	DRB1*01	DQB1*05	not found
Haplotype 2	A*02	B*40	C*07	DRB1*03	DQB1*02	not found
Haplotype 1	A*01	B*40	C*07	DRB1*03	DQB1*02	not found
Haplotype 2	A*02	B*08	C*03	DRB1*01	DQB1*05	not found
Haplotype 1	A*01	B*40	C*07	DRB1*03	DQB1*05	not found
Haplotype 2	A*02	B*08	C*03	DRB1*01	DQB1*02	not found
Haplotype 1	A*01	B*40	C*07	DRB1*01	DQB1*02	not found
Haplotype 2	A*02	B*08	C*03	DRB1*03	DQB1*05	not found
Haplotype 1	A*01	B*40	C*07	DRB1*01	DQB1*05	not found
Haplotype 2	A*02	B*08	C*03	DRB1*03	DQB1*02	not found

Haplotype 1	A*01	B*40	C*03	DRB1*03	DQB1*02	not found
Haplotype 2	A*02	B*08	C*07	DRB1*01	DQB1*05	not found
Haplotype 1	A*01	B*40	C*03	DRB1*03	DQB1*05	not found
Haplotype 2	A*02	B*08	C*07	DRB1*01	DQB1*02	not found
Haplotype 1	A*01	B*40	C*03	DRB1*01	DQB1*02	not found
Haplotype 2	A*02	B*08	C*07	DRB1*03	DQB1*05	not found
Haplotype 1	A*01	B*40	C*03	DRB1*01	DQB1*05	not found
Haplotype 2	A*02	B*08	C*07	DRB1*03	DQB1*02	not found

D. Search for [A*02-B*40-C*07 | DRB1*01-DQB1*05]:

Haplotype 2A	**A*02:01**	**B*40:01**	**C*03:04**	DRB1*13:02	DQB1*13:02	0.010
Haplotype 2B	A*03:01	B*35:01	C*04:01	**DRB1*01:01**	**DQB1*05:01**	0.013

Merge haplotype 2A and haplotype 2B

Haplotype 2	**A*02:01**	**B*40:01**	**C*03:04**	**DRB1*01:01**	**DQB1*05:01**	0.00013

E. Merge haplotype 1 and haplotype 2

Haplotype 1	A*01:01	B*08:01	C*07:01	DRB1*03:01	DQB1*02:01	0.074
Haplotype 2	A*02:01	B*40:01	C*03:04	DRB1*01:01	DQB1*05:01	0.00013
Genotype:	A*01:01	B*08:01	C*07:01	DRB1*03:01	DQB1*02:01	0.00000962
	A*02:01	B*40:01	C*03:04	DRB1*01:01	DQB1*05:01	
Normalize frequency:						
	A*01:01	B*08:01	C*07:01	DRB1*03:01	DQB1*02:01	1.0
	A*02:01	B*40:01	C*03:04	DRB1*01:01	DQB1*05:01	

Supplementary material 1: Simplified example of the calculations when linkage breakdown between HLA loci occurs. (A) The following serological split level HLA typing is used as an example: A1, A2, B8, B60, Cw7, Cw10, DR17, DR1, DQ2, DQ5. The simplified used HLA haplotype table, including the haplotype frequencies is displayed in (B). (C) First, the algorithm translates the serological split level HLA typing input into molecular equivalents using the IMGTs translation file of Steve Marsh (version: IPD-IMGT/HLA 3.24.0; available via http://hla.alleles.org/wmda/rel_dna_ser.txt). These molecular equivalents are subsequently used to setting up all potential two-field resolution HLA haplotype pairs (HLA-A, -B, -C, -DRB1, and -DQB1) that yield the given input serological split level HLA typing. In this example, no

phasing constellation was found. However, in one case only a single haplotype was found in the HLA frequency table: A*01:01, B*08:01, C*07:01, DRB1*03:01, DQB1*02:01. (D) To obtain the second HLA haplotype, the search is broadened by removing a link between HLA loci: A-B-C | DRB1-DQB1. Haplotype 2A and haplotype 2B are merged and its' frequency is calculated by multiplying the frequency of haplotype 2A with the frequency of haplotype 2B. (E) Haplotype 1 and 2 are merged and their absolute frequency is calculated by multiplying the frequency of haplotype 1 with the frequency of haplotype 2. The resulting absolute frequency is subsequently normalized as described in the materials and methods section.
